# Illness progression in older‐age bipolar disorder: Exploring the applicability, dispersion, concordance, and associated clinical markers of two staging models for bipolar disorder in an older population

**DOI:** 10.1002/gps.5816

**Published:** 2022-10-07

**Authors:** Afra van der Markt, Alexandra J. M. Beunders, Nicole C. M. Korten, Sigfried N. T. M. Schouws, Aartjan T.F. Beekman, Ralph W. Kupka, Ursula Klumpers, Annemiek Dols

**Affiliations:** ^1^ Department of Psychiatry Amsterdam UMC location Vrije Universiteit Amsterdam Amsterdam The Netherlands; ^2^ GGZ In Geest Mental Health Care Amsterdam The Netherlands; ^3^ Amsterdam Public Health Mental Health Program Amsterdam The Netherlands; ^4^ Amsterdam Neuroscience, Compulsivity, Impulsivity and Attention Program Amsterdam The Netherlands

**Keywords:** aging, bipolar disorder, illness progression, OABD, older age bipolar disorder, staging

## Abstract

**Objectives:**

The validity and applicability of two existing staging models reflecting illness progression have been studied in bipolar disorder (BD) in adults, but not in older adult populations. Staging model A is primarily defined by the number and recurrence of mood episodes, model B is defined by the level of inter‐episodic functioning. This study aimed to explore the applicability, dispersion, and concordance of, and associations with clinical markers in these two staging models in older‐age bipolar disorder (OABD).

**Methods:**

Using cross‐sectional data from the Dutch Older Bipolars study, OABD outpatients (*N* = 126, ≥50 years) were staged using models A and B. Dispersion over the stages and concordance between the models were assessed. Associations were explored between model stages and clinical markers (familial loading, childhood abuse, illness duration, episode density, treatment resistance, Mini‐Mental State Examination, and composite cognitive score).

**Results:**

Ninety subjects could be assigned to model A, 111 to model B, 80 cases to both. The majority (61%) had multiple relapses (model A, stage 3C) but were living independently (model B, stage I‐III). Concordance between models was low. For model A, the markers childhood abuse, illness duration, and episode density significantly increased over subsequent stages. Model B was not associated with a significant change in any marker.

**Conclusions:**

Assigning stages to OABD subjects was possible for both models, with age‐related adjustments for model B. Model B as currently operationalized may be less suitable for OABD or may measure different aspects of illness progression, reflected by its low correspondence with model A and lack of associated clinical markers.

## Key points

- Assigning stages to OABD subjects was possible for both models with age‐related adjustments for the model based on functioning.
- Dispersion over the stages was sufficient. Using model A, the majority (61%) of subjects concentrated in stage 3C (multiple relapses, *N* = 51); subcategories improved dispersion. Using model B, most subjects concentrated in stages I and II. Overall, the majority of subjects had multiple relapses but were still living autonomously.
- Concordance between model A (based on number of episodes) and model B (based on inter‐episodic functioning) was low.
- For model A, the markers childhood abuse, illness duration, and episode density significantly increased over subsequent stages. Illness progression according to model B was not associated with a significant change in any marker.

## 1 INTRODUCTION

The world's population is aging rapidly, but psychiatric research has made few efforts to adjust to this change. The International Society for Bipolar Disorder (ISBD) task force on Older‐Age Bipolar Disorder (OABD) defined OABD as bipolar disorder (BD) in individuals aged ≥50 years, characterized by a reduced lifespan and increased somatic comorbidity compared to individuals of the same age without BD.^1^ OABD patients are commonly excluded from studies due to stringent inclusion criteria. Since most clinical guidelines do not contain specific recommendations for older patients it remains unclear whether treatments investigated in younger adults are equally effective in OABD.^2^ There is a need for studies that focus on the OABD population to improve diagnostic precision and to develop evidence‐based procedures for this group.

Staging models provide a way to improve diagnostic precision by categorizing patients with a similar illness progression. Current diagnostics consist of a psychiatric formulation including a psychiatric classification. Staging could add to this because it allows for brief and efficient clinical communication. Moreover, staging better reflects the broad range of clinical expressions in BD and the concept of illness progression. Ultimately, staging may guide a personalized treatment approach, for example, choice of pharmacotherapy, psychotherapy and psychosocial support. Two staging models have received the most attention in BD; ‘Model A’ by Berk et al.^3^ and ‘Model B’ by Kapczinski et al.^4^ (Table 1). Model A contains five stages (0–4) and is mainly defined by the recurrence of episodes, ranging from at‐risk to chronicity. Model B describes a latent period and four stages (I‐IV) based upon inter‐episodic functional impairment, ranging from full inter‐episodic recovery to dependency in daily functioning. The validity and applicability of these staging models have been demonstrated for a general adult population with BD,^5^, ^6^, ^7^, ^8^, ^9^, ^10^ but no studies addressed whether these models can be applied directly or should be adjusted to fit an OABD sample given a potential interaction between aging and staging.

Several studies on staging in BD investigated associations between clinical markers and illness progression. A previous study by our group in adults reported familial loading, childhood abuse, earlier onset, longer illness duration, psychiatric comorbidity, and treatment resistance to be associated with model A, whereas childhood abuse, psychiatric comorbidity, cognitive functioning, and treatment resistance were related to model B.^7^ Other studies found familial loading and childhood abuse to be associated with an increased risk of developing BD or a worse outcome.^11^, ^12^

**TABLE 1:** Definition of two staging models in the current study

Staging model A by Berk et al.,^3^ adjusted by van der Markt et al.^5^		
Stage 0		Increased risk of severe mood disorder (e.g., family history, abuse, substance use).
		No specific symptoms currently
Stage 1		Mild or non‐specific symptoms of mood disorder and prodromal features: Ultra‐high risk
Stage 2		First threshold mood episode (depressive or manic)
Stage 3		Recurrence of any depressive, hypomanic, or manic/mixed episode
	A	Recurrence of sub‐threshold mood symptoms
	B	First threshold relapse
	C	Multiple relapses ≤5 episodes
		Multiple relapses 6–10 episodes
		Multiple relapses >10 episodes
Stage 4		Persistent unremitting illness; chronic (>1 year) depressive, manic or mixed episodes or rapid cycling

Staging model B by Kapczinski et al.^4^		
Latent stage		At risk for developing BD, positive family history, mood or anxiety symptoms without criteria for threshold BD
Stage I		Well‐defined periods of euthymia without overt psychiatric symptoms
Stage II		Symptoms in inter‐episodic periods related to comorbidities
Stage III		Marked impairment in cognition and functioning
Stage IV		Unable to live autonomously due to cognitive and functional impairment

The current study aimed to explore the dispersion, concordance, and associated clinical markers of two staging models for OABD patients.

First, the applicability of the two models was assessed. We checked whether it was possible to directly assign stages to the OABD subjects or whether it was necessary to first adjust the criteria to fit the OABD sample. Second, the dispersion of subjects across stages in both models was assessed. A lack of dispersion means that considerable grouping of subjects occurs within one stage. As we found a clustering of subjects in stage 3C in an earlier study in adults of all ages,^5^ resulting in a lack of dispersion over the stages, we hypothesized a lack of dispersion in the current older‐aged sample as well. We hypothesized that clustering would appear in the higher stages (i.e. more disease progression), as OABD patients often have a long illness duration. This would require additional categories in model A, stage 3C (Table 1) on top of the three established subcategories to improve dispersion. Third, we explored the concordance of both models. These two models have been developed with the aim to capture different aspects of illness progression. Therefore, we hypothesized to find a low concordance, as this would reflect that the specific concept measured by the models is distinctive. Fourth, we explored associations between the model stages and various clinical markers. We assessed vulnerability markers that drive the illness before onset: childhood abuse and familial loading. We also examined surrogate markers for illness progression: illness duration, episode density, treatment resistance, and cognitive performance. We hypothesized that model A would be associated with different clinical markers than model B, as was previously found in younger adults.^7^

## 2 METHODS

### 2.1 Participants

Data were acquired from the Dutch Older Bipolars (DOBi) dynamic cohort study.^13^ The cohort consists of OABD patients, who were receiving outpatient mental health care in Amsterdam at the time of inclusion. Exclusion criteria were: unable to communicate in Dutch or English, mental disability (IQ < 70), severe cognitive impairment (MMSE <18), or highly unstable psychiatric illness. A definitive BD diagnosis was established with MINI‐plus,^14^ based on DSM IV‐TR criteria. Patients with Bipolar I Disorder (BD‐I), Bipolar II Disorder (BD‐II), or BD Not Otherwise Specified (BD‐NOS) were included. All participants gave written informed consent. For the current study, we used data collected in 2017–2018 (wave 2). Patients were aged ≥50 years (*N* = 126, see Appendix for flowchart). The study was approved by the Medical Ethics Committee, VU University Medical Center, Amsterdam, The Netherlands.

### 2.2 Data collection DOBi study

#### 2.2.1 Diagnostic interview

During the diagnostic interview, MINI‐Plus^14^ was conducted and Global Assessment of Functioning was scored (scale 0–100; DSM‐IV, APA). The following variables were drawn from MINI‐Plus: age of onset (first episode: mania, hypomania, or depression), number of depressive and manic/hypomanic episodes, presence of rapid cycling, and current diagnosis of any addiction or anxiety disorder. The number of depressive and manic/hypomanic episodes was truncated at 50. The variable late‐onset (first episode ≥50 years) was created from the age of onset of the first episode.

#### 2.2.2 Self‐report questionnaire

The following variables were extracted from the self‐report questionnaire for Bipolar Disorders (QBP‐NL^15^), an adaptation and Dutch translation of the Enrolment Questionnaire from the Stanley Foundation Bipolar Network^16^, ^17^: sociodemographic data (age, gender, level of education, marital status, occupation status); number of psychiatric admissions; familial loading; abuse during childhood or adolescence; and inter‐episodic functioning. Other self‐report questionnaires assessed history of psychotic features and the Centre for Epidemiologic Studies Depression scale (CES‐D^18^; scale 0–60).

#### 2.2.3 Neuropsychological assessment

Neuropsychological assessment included 13 neuropsychological tests, Mini‐Mental State Examination (MMSE^19^), and Young Mania Rating Scale (YMRS, scale 0–60^20^).

#### 2.2.4 Physical health interview

A physical health interview was conducted by a trained doctor or research assistant, identifying the total number of somatic comorbidities (out of 15), current alcohol use, and recreative drug use.

Detailed information on data collection, measurement, operationalization, and definitions of all investigated variables are available in previous publications from our group.^13^, ^21^

Application of staging models A and B to the OABD study sample.

**FIGURE 1:**
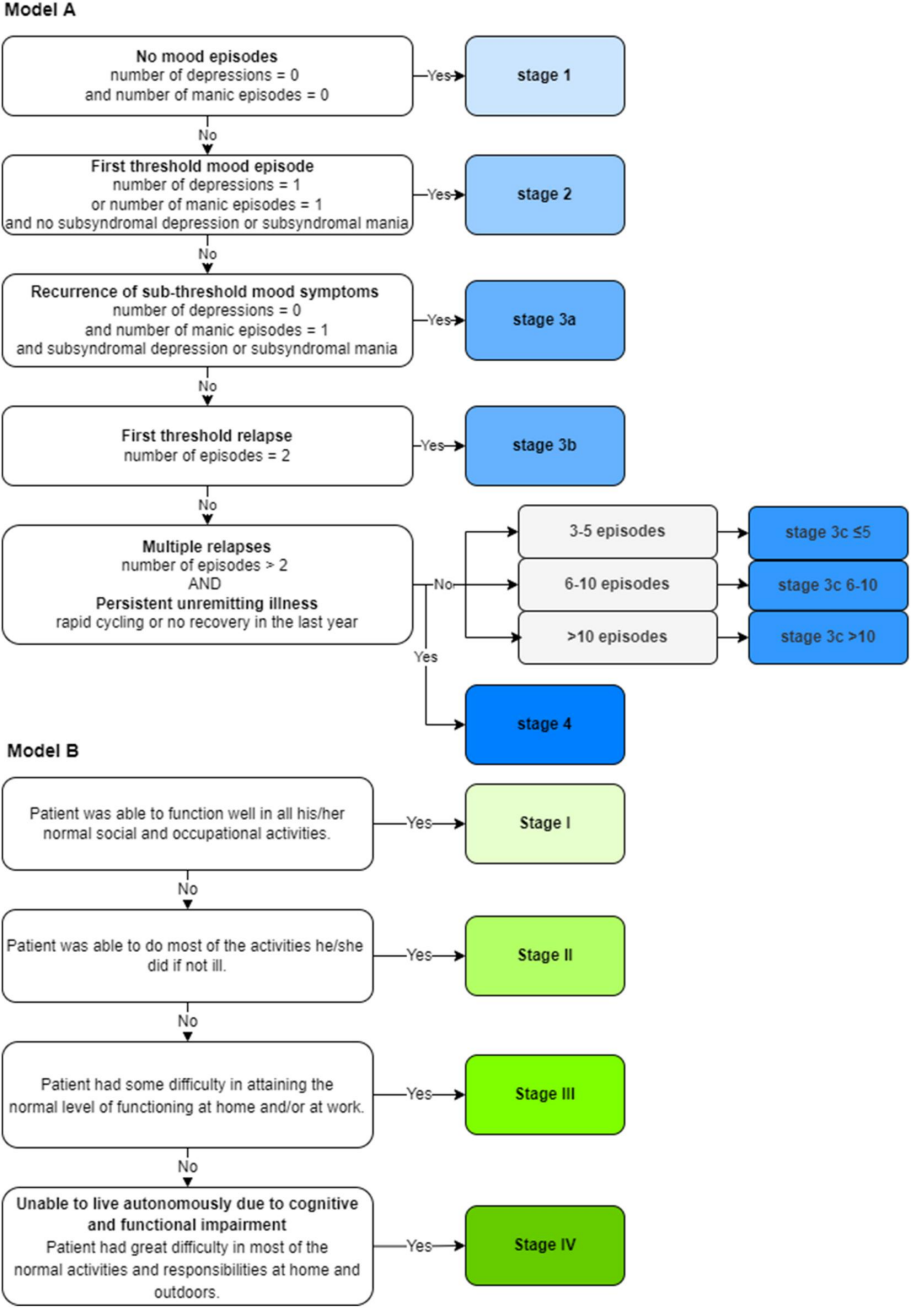
Application of the staging models to the Dutch Older Bipolars (DOBi) study population

All subjects were staged according to staging models A and B (see Figure 1).

#### 2.2.5 Staging model A

For staging according to model A, we used the lifetime number of mood episodes, the rapid cycling specifier, subthreshold mood symptoms, and the presence of chronic bipolar illness. Stages 0 and 1 were not present since all subjects were diagnosed with BD. A lifetime history of one mood episode was classified as stage 2 or 3a, depending on the subsequent presence of subthreshold mood symptoms, defined as a CES‐D score of 14 or 15^22^ or a YMRS score of 14‐19.^23^ A history of two mood episodes led to stage 3b. A history of multiple mood episodes without rapid cycling or chronic bipolar illness led to stage 3c. Similar to our previous publication,^5^ we observed it was necessary to further subdivide into substages with cutoffs at 5 and 10 mood episodes to improve dispersion.

We adjusted the definition of chronicity from the ISBD staging task force from at least 2 years^24^ to 1 year due to data availability. Stage 4 was considered present if the QBP‐NL item on inter‐episodic functioning (‘How did the patient function IN BETWEEN EPISODES in the past year, compared to their normal level of functioning?’) was answered with E.‘It was not possible to distinguish a period in between episodes of depression or hypomania or mania (i.e. constantly alternating between episodes, or a depressive, hypomanic or manic episode for the full past year’).

#### 2.2.6 Staging model B

In our previous studies, stages from model B were assigned based on the ability to work and function autonomously.^5^, ^7^ Since these items may not reflect the impact of illness progression in older age, an alternative strategy was developed based on expert consensus. Each participant was assigned a stage in model B according to the answer to the QBP‐NL item on inter‐episodic functioning (‘How did the patient function IN BETWEEN EPISODES in the past year, compared to their normal level of functioning?’). The following answers were used to assign a stage from model B: A. ‘able to function well in his/her normal social and occupational activities’—stage I. B. ‘able to do most of the activities he/she did if not ill’—stage II C. ‘some difficulty in attaining the normal level of functioning at home and/or at work’—stage III. D. ‘great difficulty in most of the normal activities and responsibilities at home and outdoors’—Stage IV.

### 2.3 Clinical markers

Two vulnerability markers, familial loading and childhood abuse, were selected from the self‐report questionnaire (QBP‐NL^15^) because they have widely been established as influencing onset and further illness progression.^5^ Familial loading was defined as having at least one parent with a history of depression, BD, or psychosis. Childhood abuse included any type of self‐reported verbal, physical, or sexual abuse during childhood or adolescence.

Surrogate markers for illness progression were selected or computed: illness duration, episode density, treatment resistance, MMSE, and composite cognitive score. Illness duration was calculated as years between age at onset and age at assessment. Episode density was calculated by dividing the total number of mood episodes by illness duration. Treatment resistance was created from the inventory of current medication by categorizing each drug into one of four medication classes: lithium, antipsychotics, anticonvulsants, or antidepressants. Monotherapy with lithium or another mood stabilizer such as valproate is the preferred treatment according to Dutch guidelines, therefore using two or more medication classes should only be considered if monotherapy was ineffective and was considered indicative of treatment resistance since. This is in line with the treatment resistance definition by Hidago‐Mazzei et al.^25^ Benzodiazepines were not considered indicative of treatment resistance. Two variables for cognitive performance were used: MMSE and a ‘composite cognitive score’ based on a full neuropsychological assessment, calculating the mean of 13 neuropsychological test Z‐scores.^21^ The composite cognitive score was corrected for age and education level.

### 2.4 Statistical analysis

Data were analyzed using SPSS 27.^26^ Descriptive characteristics were reported with mean (SD), range or % [N]. A cross‐table was constructed to assess the dispersion of subjects across model stages. Concordance between the two models was calculated with Kendall's tau‐b (τb) correlation coefficient (Two‐tailed, with stage 3c of model A subdivided into categories with cut‐offs at 5 and 10 mood episodes) and Cohen's weighted kappa (using the main stages 1–4 for model A and I—IV for model B). Differences in clinical markers between the stages were evaluated with the Kruskal‐Wallis test for continuous variables and the Chi‐squared test (trend) for categorical variables.

## 3 RESULTS

### 3.1 Descriptive characteristics of the study sample

Socio‐demographics, psychiatric characteristics, comorbidities, psychotropic medication use, and cognitive performance of the total sample (*N* = 126) are listed in Table 2. Mean age was 64.5 years (SD 7.7, range 51.3–86.8); 56.3% were female, 55.3% had a high education level, and 35.4% had a current job. The sample consisted of BD‐I (42.9%), BD‐II (56.3%), and BD‐NOS (0.8%). Average age of onset was 29.9 years (SD 15.6); 13.5% had a late illness onset (age ≥50). Mean illness duration was 34.6 years (SD 14.7, range 0.8–66.8).

**TABLE 2:** Characteristics of the study sample (*N* = 126)

	Total N	Mean (SD), range or % [*N*]
Socio‐demographics		
Age, in years	126	64.5 (7.7), 51.3–86.8
Gender, female	126	56.3% [71]
Level of education, high	114	55.3% [63]
Relationship status, married or with steady partner	114	58.8% [67]
Occupational status, current paid job	113	35.4% [40]
Psychiatric characteristics		
Bipolar disorder subtype	126	
BD‐I		42.9% [54]
BD‐II		56.3% [71]
BD‐NOS		0.8% [1]
YMRS, scale 0–60	96	3.2 (3.6), 0–18
CES‐D, scale 0–60	104	14.5 (12.3), 0–51
Age of onset, in years	126	29.9 (15.6), 4–76
Late onset, ≥50 years	126	13.5% [17]
Illness duration, in years	126	34.6 (14.7), 0.8–66.8
Number of manic/hypomanic episodes	112	8.9 (11.8), 1–50
Number of depressive episodes	95	12.70 (13.7), 1–50
Episode density,^a^ in nr of episodes per year	93	2.43 (7.18), 0–51
Psychiatric admissions, five or more	112	14.3% [16]
Psychotic features, lifetime	124	54.8% [68]
Familial loading	108	
One parent		36.1% [39]
Two parents		7.4% [8]
Abuse during childhood or adolescence	112	59.8% [67]
GAF score, scale 0–100	121	64.8 (14.0), 40–100
Comorbidities		
Diagnosis of any addiction, current	124	8.9% [11]
Diagnosis of any anxiety disorder, current	124	16.9% [21]
Nr. of somatic comorbidities (out of 15)	124	2.2 (1.5), 0–8
Current psychotropic medication use		
Lithium	122	54.1% [66]
Antipsychotics	105	41.9% [44]
Anticonvulsants	119	26.9% [32]
Antidepressants	104	28.8% [30]
Benzodiazepines	104	36.5% [38]
Treatment resistance^b^	119	42.9% [54]
Cognitive performance		
MMSE, scale 0–30	101	28.2 (1.7), 23–30
Composite cognitive score^c^	109	0.094 (0.58)

*Notes*: Benzodiazepines were not used for the calculation of treatment resistance. Subjects were divided into those with less than two medication classes and those with two or more medication classes.

Abbreviations: CES‐D, the Centre for Epidemiologic Studies Depression scale; GAF, Global Assessment of Functioning; MMSE, Mini‐Mental State Examination; NOS, not otherwise specified; YMRS, Young Mania Rating Scale.

^a^ Episode density was calculated by dividing illness duration by total number of mood episodes.

^b^ The variable treatment resistance was created from the inventory of current medication.

^c^ Mean Z‐score from 13 neuropsychological tests, see Beunders et al. (22) for the procedure.

### 3.2 Applicability and dispersion

#### 3.2.1 Model A

Using model A, 74% of the study sample (*N* = 90) could be assigned to a stage (Figure 1). Non‐allocations were due to missing data. Participants with stage 0 (at risk) or stage 1 (prodromal) were not present, as a clinical sample was used. Five subjects were assigned to stage 2 (threshold episode), three to stage 3B (first threshold relapse), 9 to stage 3C ≤ 5 episodes, 116 to stage 3C 6–10 episodes, 30 to stage 3C > 10 episodes (multiple relapses), and 27 to stage 4 (multiple relapses and chronic illness or rapid cycling). No subjects were assigned to stage 3A (subthreshold mood symptom recurrence).

#### 3.2.2 Model B

For model B, a stage could be appointed to 87.3% of the sample (*N* = 111) (Figure 1). Non‐allocations were due to missing data. No subjects were classified as latent stage as the sample only consisted of subjects with BD, 46 subjects were assigned to stage I (inter‐episodic euthymia without symptoms), 39 to stage II (inter‐episodic symptoms), 21 to stage III (marked inter‐episodic impairment), and five to stage IV (inability to live autonomously).

**FIGURE 2:**
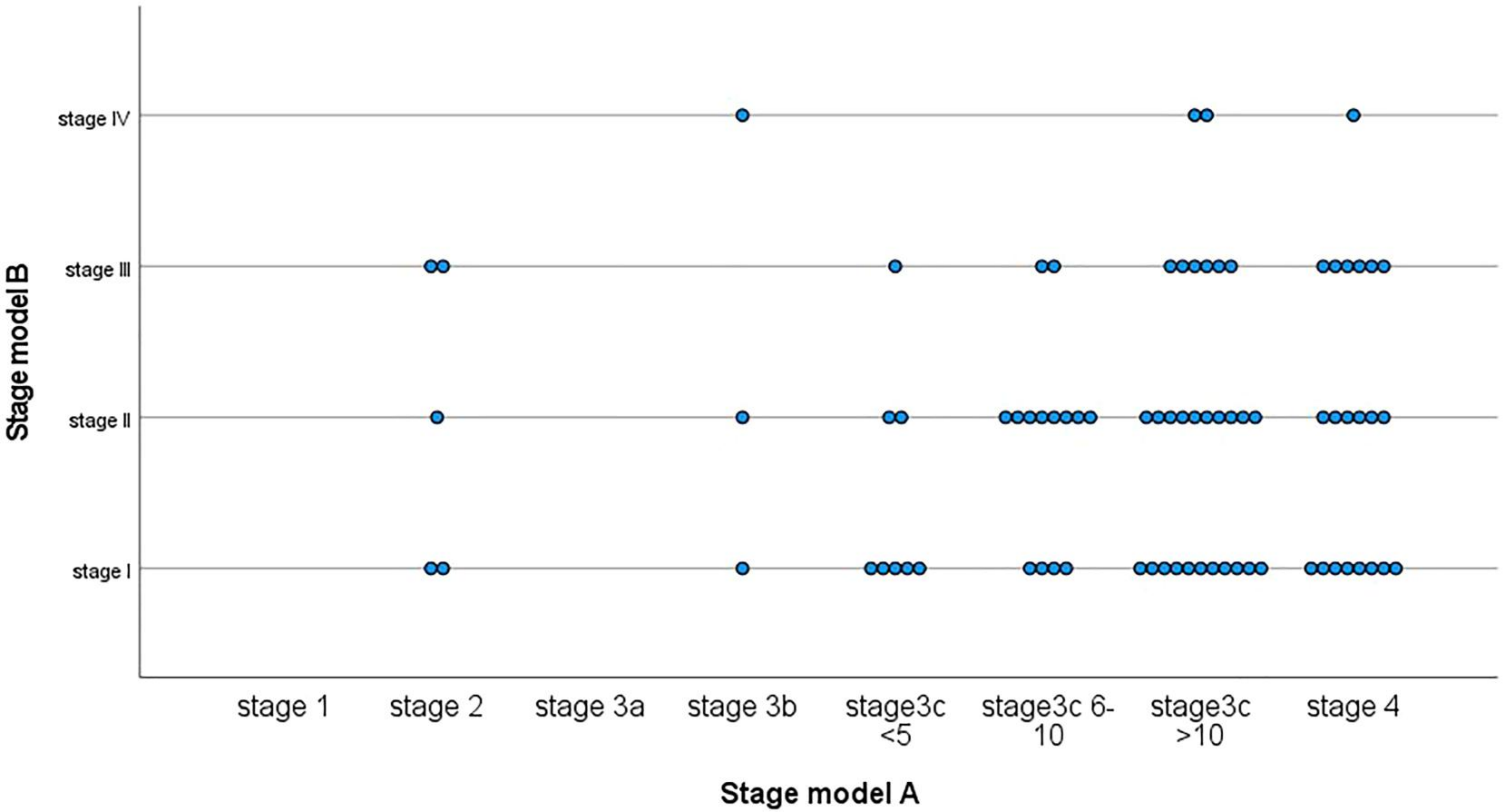
Distribution of subjects over the stages of both models

80 subjects could be assigned to both models. Table 3 shows the dispersion of these subjects over the two stages. Using model A, the majority (61%) of subjects clustered in stage 3C (multiple relapses, *N* = 51); adding subcategories improved dispersion. Using model B, most subjects concentrated in stages I and II. Overall, the majority of subjects had multiple relapses but were still living autonomously Figure 2.

**TABLE 3:** Clinical markers for illness progression per stage of both staging models

	Model A								Model B				
	Stage 2	Stage 3a	Stage 3b	Stage 3c < 5 episodes	Stage 3c 6–10 episodes	Stage 3c > 10 episodes	Stage 4	Chi‐squared (trend) or Kruskal‐Wallis test	Stage I	Stage II	Stage III	Stage IV	Chi‐squared (trend) or Kruskal‐Wallis test
Total N in stage	5	0	3	9	16	30	27		46	39	21	5	
Vulnerability markers													
Familial loading % [*N*]	40.0% [2]	‐	0% [0]	37.5% [3]	50.0% [7]	42.9% [12]	61.9% [13]	*X* ^2^(5) = 5.2, *p* = 0.396	67.5% [27]	53.1% [17]	44.4% [8]	40.0% [2]	*X* ^2^(3) = 3.7, *p* = 0.291
Childhood abuse % [*N*]	20.0% [1]	‐	0% [0]	50.0% [4]	53.3% [8]	62.1% [18]	81.8% [18]	*X* ^2^(5) = 12.8, *p* = 0.025	60.5% [26]	50.0% [16]	66.7% [2]	80.0% [5]	*X* ^2^(3) = 2.5, *p* = 0.482
Surrogate markers of illness progression													
Treatment resistance % [*N*]	20.0% [1]	‐	33.3% [1]	44.4% [4]	40.0% [6]	58.6% [17]	45.8% [11]	*X* ^2^(20) = 20.9, *p* = 0.400	34.9 [15]	51.4 [19]	60.0 [12]	40.0 [2]	*X* ^2^(12) = 17.0, *p* = 0.151
Illness duration, in years mean (SD)	9.6 (8.3)	‐	5.5 (6.4)	27.1 (10.5)	35.8 (10.1)	42.8 (11.3)	35.7 (14.8)	H(5) = 30.1, *p* < 0.001	36.7 (15.2)	34.5 (13.9)	33.2 (14.8)	41.1 (16.1)	H(3) = 2.3, *p* = 0.522
Episode density, episodes per year mean (SD)	0.9 (0.7)	‐	1.6 (1.8)	0.2 (0.1)	0.2 (0.1)	0.6 (0.3)	3.0 (4.1)	H(5) = 41.6, *p* < 0.001	4.6 (10.8)	1.6 (4.9)	0.9 (1.1)	0.6 (0.3)	H(3) = 2.0, *p* = 0.581
MMSE score mean (SD)	29.3 (0.6)	‐	28.5 (0.7)	27.6 (2.6)	29.3 (1.0)	28.1 (1.5)	28.3 (1.5)	H(5) = 7.4, *p* = 0.192	28.5 (1.8)	28.0 (1.7)	28.3 (1.7)	27.5 (1.3)	H(3) = 4.6, *p* = 0.205
Composite cognitive score mean (SD)	−0.1 (0.3)	‐	0.0 (0.3)	0.3 (0.3)	0.2 (0.4)	−0.1 (0.3)	0.1 (0.4)	H(5) = 9.9, *p* = 0.080	0.0 (0.3)	0.0 (0.4)	0.1 (0.4)	0.1 (0.2)	H(3) = 0.5, *p* = 0.923

*Notes*: MMSE, Mini‐Mental State Examination. See text for definitions of the variables. Given that the sample consisted of subjects with manifest BD, stages 0 and 1 of model A, and latent stage of model B were omitted in the table.

### 3.3 Concordance

The correlation between models was low and not statistically significant (Kendell's tau, τb = 0.059, *p* = 0.536). Cohen's weighted kappa showed no agreement between models (*κ* = −0.13, 95%CI = −0.062 to 0.035, *p* = 0.576).

### 3.4 Associations with clinical markers

#### 3.4.1 Vulnerability markers

In model A, childhood abuse was significantly associated with higher stages (*X* ^2^(5) = 12.8, *p* = 0.025), but familial loading was not. In model B, childhood abuse and familial loading were not related to higher stages (Table 3).

#### 3.4.2 Surrogate markers for illness progression

In model A, a higher stage was significantly associated with longer illness duration (H(5) = 30.1, *p* < 0.001) and higher episode density (H(5) = 41.6, *p* < 0.001), but not in model B. MMSE and composite cognitive score were not significantly associated with change over the stages of either model (Table 3).

## 4 DISCUSSION

In our sample of 126 outpatients with OABD, assigning stages was possible for the majority of subjects according to two staging models designed for BD in individuals of any age. The majority of subjects had multiple relapses of mood episodes (higher stages in model A) but retained a high level of inter‐episodic functioning (lower stages in model B). Dispersion over the stages was acceptable after adding subcategories of stage 3c for model A. We found a low concordance between both models. In model A, the chance of being in a higher stage was associated with childhood abuse, longer illness duration, and episode density. In contrast, in model B, progression to a higher stage was not associated with any clinical marker.

### 4.1 Applicability

As in our previous study with a general adult sample,^5^ it was possible to assign stages to patients for both models in our OABD sample. We hypothesized that it would be necessary to adjust the criteria to fit the OABD sample, which was true for model B but not for model A. For model B, Stage III is defined as ‘marked impairment in cognition and functioning’.^4^ However, around the fifth or sixth decade, functioning or ‘working hours’ may be reduced due to physical impairments or the desire to experience more leisure time. Stage IV is defined as being unable to live autonomously due to cognitive and functional impairment.^4^ Beyond the age of 60, age itself and the co‐occurring physical impairments may considerably influence the ability to function autonomously. Therefore, instead of using data on the ability to work and/or live independently, we used data on general inter‐episodic functioning to stage subjects with model B.

### 4.2 Dispersion

We hypothesized that clustering would appear in the higher stages, due to illness progression, as OABD patients often have a long illness duration. There was no considerable grouping in one stage or substage of either staging model. The application of both models to the OABD thus led to sufficient dispersion of the subjects over the stages of this model. Further subdivision of stage 3C of model A due to clustering in one substage was not required after adopting the suggested subcategories for stage 3C.^5^

### 4.3 Concordance

Concordance between the two models was low, as both Kendall's tau‐b and Cohen's weighted Kappa showed low to no agreement between models. This suggests that both staging models reflect different concepts, which was to be expected as both models were developed to capture different aspects of illness progression. Therefore, in OABD patients, we suggest using both models complementarily, as was previously suggested for subjects of all ages.^7^ Alternatively, the lack of concordance may suggest that model B, operationalized in the current study as inter‐episodic functioning, not only reflects illness progression. This needs to be explored in further studies which should try to improve the current operationalization.

## 5 ASSOCIATED CLINICAL MARKERS

As hypothesized, we found different associated markers for each model, adding to the idea that both models measure different aspects of illness progression. Two surrogate markers of illness progression were associated with an increased chance of higher stages for model A. Higher stages were associated with a longer illness duration. This finding seems intuitive since patients with longer illness duration had more time to develop subsequent mood episodes. This is in line with our study of bipolar patients of all ages.^7^ Interestingly, model A was also associated with a higher episode density, suggesting that mood episodes succeed one another more rapidly in higher stages of model A. Reinares et al.^28^ also found an association between increased episode density and illness progression. These findings are in agreement with the sensitization and kindling hypothesis by Post^29^ that postulates that each experienced episode reduces the threshold for recurrence, leading to a higher recurrence rate and thus increased episode density or even progression towards rapid cycling or chronicity.

In model A, treatment resistance was not associated with an increased chance of higher stages. This is in contrast to our earlier study,^7^ where we found that treatment resistance was significantly related to higher stages of model A. This may be explained by differences in sampling between these studies. In the earlier study, medication use was higher compared to our current OABD sample (lithium 93.3% vs. 54.1%, antipsychotics 74.5% vs. 41.9%, antidepressants 52.9% vs. 28.8%, anticonvulsants 28.3% vs. 26.9%). Also, the earlier study only included BD‐I patients whereas the current study included BD‐I, BD‐II, and BD‐NOS patients. The earlier study cohort was recruited from several clinical and non‐clinical settings while our OABD cohort consisted of patients who received care at one outpatient specialized center. Alternative explanations for the discrepancy may be that the sample size was too small to detect significant differences or that patients may have learned to manage their bipolar illness better with advancing age without the need for medication.^30^

Regarding model B, the chance of being in a higher stage was not associated with any of the investigated clinical markers. For illness duration, our previous the study^7^ also did not find a relation with model B. However, other markers were associated with model B in various studies, including childhood abuse,^7^ familial loading,^7^ and treatment resistance.^7^, ^9^, ^10^ This suggests that model B as currently operationalized may be less suitable for OABD. Future studies should explore other measurements for inter‐episodic functioning for staging with model B in OABD, such as the Functioning Assessment Short Test (FAST‐O^31^) which has already been validated in OABD patients.

An unanticipated finding was that cognitive performance was not associated with an increased chance of being in higher stages of either model. There may be several explanations. In older adults, cognitive performance may not (further) decrease in BD over the illness course, a hypothesis supported by evidence from several longitudinal studies in OABD.^32^, ^33^, ^34^ Also, models A and B may not be sensitive enough to measure a change in cognitive performance. Finally, this negative result may be influenced by the exclusion of subjects with an MMSE‐score under 18 (*N* = 6).

### 5.1 Strengths and limitations

The main strength of this study is that to our knowledge this is the first attempt to apply staging models to an OABD population specifically, setting a precedent for future studies. Our study has several limitations. Both staging models are based on clinical data and are therefore more subjective than a staging model based on objectively measured pathophysiological markers. Currently, no validated physical markers reflecting illness progression have been discovered, so future studies should focus on identifying such markers. As study was performed cross‐sectionally, the longitudinal course and outcome could not be assessed. The reported number of mood episodes (for model A) and self‐report of the extent of inter‐episodic functioning (for model B) may be subject to recall bias. Also, model B stages could not be assigned on the original criteria as proposed by Kapczinski et al.^4^ due to a high prevalence of retirement and physical illnesses in OABD. Instead, we used a question on general inter‐episodic functioning based on expert consensus.

### 5.2 Clinical implications and future outlook

In conclusion, our study shows that both staging models A and B can be applied to subjects with OABD, though be it with some adjustments. For model A, higher stages were associated with more childhood abuse, longer illness duration, and higher episode density, but not with familial loading, treatment resistance, or cognitive performance. For model B, higher stages were not associated with any of the markers that we used for this study and therefore further exploration of alternative staging strategies is needed.

Since both models appeared to reflect different aspects of illness progression in a previous study with a general adult sample, we suggest using both models complementarily until more research is conducted on the validity of model B in OABD. Future studies should compare these two models over the life‐span in terms of dispersion, concordance and associated markers. Ideally, a future staging model could follow the same approach as the TNM staging model for tumors,^27^ in which three distinct aspects of illness progression (tumor size, lymph node involvement, and metastases) lead to one composite classification. For staging BD, these three dimensions may include: number of episodes (model A), inter‐episodic functioning (model B), and a yet‐to‐be‐discovered biomarker indicating the extent of biological illness progression.

## CONFLICT OF INTEREST

The authors declare that there is no conflict of interest that could be perceived as prejudicing the impartiality of the research reported.

## Supporting information

Supporting Information S1

## Data Availability

The data that support the findings of this study are available on request from the corresponding author. The data are not publicly available due to privacy or ethical restrictions.
