# Supplementary material for: Illness progression in older‐age bipolar disorder: Exploring the applicability, dispersion, concordance, and associated clinical markers of two staging models for bipolar disorder in an older population
Source: Int J Geriatr Psychiatry. 2022 Oct 7;37(11):10.1002/gps.5816. doi: 10.1002/gps.5816 (PMC9828008; doi:10.1002/gps.5816)
Supplement: Supplementary file 1 — Supporting Information S1 [file GPS-37-0-s001.docx]

**Supplemental Figure 1. Flowchart for wave 2 of the DOBi dynamic cohort study (DOBi 2)**
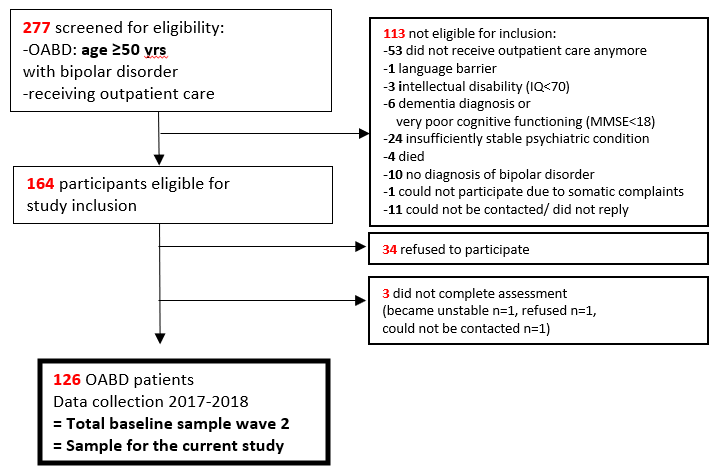


| **Supplemental Table 1.** **Dispersion** **of the study sample over the models** | | | | | | | | |
| --- | --- | --- | --- | --- | --- | --- | --- | --- |
|  | | **Model B** | | | |  |  | |
|  | | Stage I | Stage II | Stage III | Stage IV |  | | *Model A only,*  *no stage for model B* |
| **Model A** | | Periods of Euthymia | Inter-episodic symptoms | Marked impairment | Unable to live autonomously | ***Total*** | |  |
| Stage 1 | Prodromal | 0 | 0 | 0 | 0 | 0 | | 0 |
| Stage 2 | Threshold episode | 2 | 1 | 2 | 0 | **5** | | 0 |
| Stage 3A | Subthreshold recurrence | 0 | 0 | 0 | 0 | **0** | | 0 |
| Stage 3B | First threshold relapse | 1 | 1 | 0 | 1 | **3** | | 0 |
| Stage 3C | Multiple relapses | 20 | 20 | 9 | 2 | **51** | | 4 |
|  | ≤5 episodes | 5 | 2 | 1 | 0 | **8** | | 1 |
|  | 6–10 episodes | 4 | 8 | 2 | 0 | **14** | | 2 |
|  | >10 episodes | 11 | 10 | 6 | 2 | **29** | | 1 |
| Stage 4 | Persistent unremitting illness | 8 | 6 | 6 | 1 | **21** | | 6 |
|  | ***Total*** | **31** | **28** | **17** | **4** | **80** | |  |
| *Model B only, no stage for model A* | | 15 | 11 | 4 | 1 |  | |  |
| ***Notes****: Given that the sample consisted of subjects with manifest BD, stages 0 and 1 of model A, and latent stage of model B were omitted in the table.* | | | | | | | | |
